# Genetic Factors That Contribute to Antibiotic Resistance through Intrinsic and Acquired Bacterial Genes in Urinary Tract Infections

**DOI:** 10.3390/microorganisms11061407

**Published:** 2023-05-26

**Authors:** Mohammed Harris, Tracy Fasolino, Diana Ivankovic, Nicole J. Davis, Noel Brownlee

**Affiliations:** Department of Healthcare Genetics and Genomics, Clemson University, Clemson, SC 29634, USA

**Keywords:** antibiotic resistance genes, urinary tract infection, bacterial genetics, molecular diagnostics, antimicrobial resistance genes, polymicrobial infections, UTI, multiplex PCR

## Abstract

The overprescribing and misuse of antibiotics have led to the rapid development of multidrug-resistant bacteria, such as those that cause UTIs. UTIs are the most common outpatient infections and are mainly caused by *Escherichia coli* and *Klebsiella* spp., although some Gram-positive bacteria, such as *Pseudomonas aeruginosa*, have been isolated in many cases. The rise of antimicrobial-resistant bacteria is a major public health concern, as it is predicted to lead to increased healthcare costs and poor patient outcomes and is expected to be the leading cause of global mortality by 2050. Antibiotic resistance among bacterial species can arise from a myriad of factors, including intrinsic and acquired resistance mechanisms, as well as mobile genetic elements, such as transposons, integrons, and plasmids. Plasmid-mediated resistance is of major concern as drug-resistance genes can quickly and efficiently spread across bacterial species via horizontal gene transfer. The emergence of extended-spectrum β-lactamases (ESBLs) such as *NDM-1*, *OXA*, *KPC*, and *CTX-M* family members has conferred resistance to many commonly used antibiotics in the treatment of UTIs, including penicillins, carbapenems, cephalosporins, and sulfamethoxazole. This review will focus on plasmid-mediated bacterial genes, especially those that encode ESBLs, and how they contribute to antibiotic resistance. Early clinical detection of these genes in patient samples will provide better treatment options and reduce the threat of antibiotic resistance.

## 1. Antimicrobial Resistance

Antimicrobial resistance (AR) is a global threat to human health, healthcare systems, and the availability of effective treatments for deadly pathogens. AR is projected to be the leading cause of global mortality by 2050 due to the diminishing utility of current antibiotics and the lack of new antibiotics in the market [1]. Anthropogenic factors that contribute to antimicrobial resistance include overuse and misuse of antibiotics, incorrect diagnoses, and the prophylactic use of antibiotics in animal husbandry. The increase in multidrug-resistant bacterial strains is a major public health concern, as antibiotics are used routinely in clinical settings for the treatment of bacterial infections. Among the ~3 million infections caused by multidrug-resistant bacteria in the US are a growing number of urinary tract infections (UTIs) that can no longer be treated with the most common antibiotics. UTI-related symptoms lead to almost 10 million office visits and around 3 million emergency visits per year in the US [2]. UTIs are mainly caused by *Escherichia coli* and *Klebsiella* spp., although other bacteria, including Gram-positive microorganisms, such as *Pseudomonas aeruginosa*, have been isolated in some cases [2]. AR genes can disseminate quickly and efficiently across bacterial species, warranting further attention to curb the spread of resistance. To counteract AR, coordinated efforts by various healthcare settings are being made to improve the selection, dosing, duration, and route of administration of antibiotics, a concept known as antibiotic stewardship [3]. Global eradication of AR requires, at a minimum, large-scale programs that educate and inform healthcare workers and patients about the appropriate dispensation and use of antimicrobial drugs. Additionally, a better understanding of genetic factors in bacteria that contribute to AR will aid in better diagnostic screenings to provide optimal care for patients. This review will focus on various classes of antimicrobial agents, genetic resistance mechanisms that bacteria have evolved to evade them, and how this knowledge can be exploited for more effective patient care and antibiotic stewardship.

## 2. Mechanisms of AR in Bacteria

Intrinsic and acquired resistance mechanisms enable the transfer of AR in bacterial species. Intrinsic resistance is the phenomenon by which bacteria become transiently refractory to an antibiotic due to phenotypic characteristics. Intrinsic resistance to antibiotics can extend the duration of treatment, cause treatment failure, and promote the generation of acquired resistance in treated patients [4]. Acquired resistance mechanisms are those that are acquired in response to drug exposure, such as drug target modification, drug efflux, uptake reduction, and inactivation/degradation of the compound. Gram-negative bacteria have been known to use any of these mechanisms in acquiring AR, while Gram-positive bacteria are less likely to affect drug uptake or efflux [5]. Drug target modification is frequently observed across several drug classes. For instance, Gram-positive bacteria can become resistant to β-lactams by alteration of penicillin-binding proteins (PBPs), which are essential for bacterial cell wall synthesis. Bacteria can inactivate drugs either by degrading them or adding chemical modifications to them. For example, aminoglycosides are frequently inactivated by the transfer of a phosphoryl or AMP group [6]. Drug uptake can be altered via changes in porins, which are channels through which hydrophilic molecules can enter the cell. For example, resistance to carbapenems by members of *Enterobacteriaceae* is established by reducing the number of porins [7,8]. Genes for efflux pumps are chromosomally encoded and function to rid a bacterial cell of toxic substances. Some bacteria inherently possess or can acquire multidrug resistance (MDR) efflux pumps, which pump out antibiotics from the cell, making the cell resistant to its effects.

Mobile genetic elements promote intra- and intercellular DNA mobility and play a key role in AR dissemination. Several types of these elements play a role in promoting AR in bacteria, such as transposons, integrons, and plasmids. Composite transposons are mobile genetic elements consisting of two insertion sequences flanking DNA that often contain AR genes. Integrons are bacterial genetic elements able to promote the acquisition and expression of genes embedded within gene cassettes [9]. A gene cassette is a < 1 kb mobile element that contains 1–2 genes and an attC recombination site. Plasmids are extrachromosomal mobile genetic elements that are thought to drive the evolution of AR [10]. Plasmid-mediated resistance is of major concern as resistance genes can quickly and efficiently spread across bacterial species via horizontal gene transfer. Within mobile genetic elements are factors that mediate AR (Table 1) by a variety of mechanisms, some of which are discussed below. 

## 3. Bacterial Genes That Contribute to Antimicrobial Resistance

### 3.1. β-lactamases

β-lactamases make up a family of >2800 enzymes that evolved as a mechanism against naturally occurring β-lactams and have since become a focus of pharmaceutical research. These enzymes are produced by some bacteria and provide resistance to β-lactam antibiotics, such as penicillins, cephalosporins, cephamycins, and carbapenems. Since the discovery of penicillin in the early 20th century, β-lactams have played a fundamental role in the treatment of bacterial infections. This class of antibiotics is continuously undergoing development and improvement to combat the AR trend and remains the most prescribed class of antibiotics [11]. 

#### 3.1.1. AmpC β-lactamases

AmpC β-lactamases are cephalosporinases that confer resistance to many bacterial isolates, especially *Enterobacteriaceae* [12,13]. Microorganisms overexpressing AmpC β-lactamases are clinically problematic as they are usually resistant to all β-lactam drugs, except cefepime, cefpirome, and carbapenems. AmpC β-lactamase resistance can arise via chromosomally encoded ampC or by the acquisition of a plasmid or transferable genetic element with ampC. The latter mechanism leads to constitutive AmpC production, which results in increased resistance and more serious clinical outcomes. *Enterobacter* spp. and *K. aerogenes* are the top pathogens with the highest prevalence of AmpC β-lactamase induction [14]. To date, there are >180 AmpC genes across six families; detection of plasmid-mediated ampC β-lactamase gene families via multiplex PCR or other high throughput assays can aid in prescribing the proper antibiotic regimen [15,16].

CMY-2

The most common plasmidic ampC gene reported in Enterobacteriaceae, including *E. coli*, is *blaCMY-2* [17,18]. *CMY-2* plasmids are proposed to have undergone transfer between different bacterial species and may have been transmitted between livestock, such as cattle and swine, and humans [19]. This cross-species transmission is largely enabled by the spread of IncA/C and IncI1 plasmids among *E. coli* from humans, animals, and environmental sources [20,21]. Further, the chromosomal *blaCMY-2* transfer from *E. coli* into a small endogenous ColE1-like plasmid via insertion sites, ISEcp1, has been demonstrated [22].

FOX

FOX-type enzymes are plasmid-encoded AmpC β-lactamases that are especially active against cefoxitin. *FOX-1* was originally identified in *K. pneumoniae*, though it was recently shown to have evolved from *Aeromonas allosaccharophila*, a fish pathogen [23,24]. To date, 11 FOX variants have been reported.

#### 3.1.2. Carbapenemases

Carbapenems are considered one of the most effective antibacterial agents and are generally reserved for the treatment of multidrug-resistant (MDR) bacterial infections. However, with the rapid and extensive spread of carbapenem-resistant *Enterobacteriaceae*, carbapenems have become less effective against their targets. Resistance is mainly mediated by the production of carbapenemases.

##### Class A Carbapenemases

*Klebsiella pneumoniae* carbapenemases (KPC)

KPCs are the most prevalent of the class A carbapenemases and are encoded by the *blaKPC* gene. The *blaKPC* gene is carried on a mobile genetic element and confers resistance to all β-lactam agents [25]. Plasmids carrying *blaKPC* are related to resistance factors for other antibiotics, which makes them, especially concerning due to interspecies transfer, which can increase the polymicrobial infectious state [26]. *K. pneumoniae* is the most prevalent bacterial species carrying KPCs, but other Gram-negative bacilli have been shown to carry the enzyme as well [27]. Its location in the Tn4401 transposon makes the *blaKPC* gene more likely to spread across different types of Gram-negative bacteria [28]. KPC producers have been identified worldwide, and most cases have been linked to hospitalized patients [29].

##### Class B Carbapenemases

Class B carbapenemases are resistant to most β-lactamase inhibitors, are inhibited by metal chelating agents, and have one or more zinc atoms in their active site. The genes encoding many class B carbapenemases, such as those presented below, are located within a variety of integron structures and incorporated in gene cassettes [30].

(1) IMP

IMP-type carbapenemases are one of the two most widely distributed carbapenem-resistant *Enterobacteriaceae* β-lactamases, with the other being *VIM*. *IMP-1* is encoded by the transferable *blaIMP* gene and confers resistance to imipenem, a broad-spectrum intravenous β-lactamase. *IMP-1* was first detected in a *P. aeruginosa* isolate in Japan in the 1990s [31]. Since then, clinical isolates of many bacteria harboring the *IMP* genes, such as *K. pneumoniae*, *P. aeruginosa*, and *S. marcescens*, have been identified worldwide [32,33,34,35]. A clinical isolate of ertapenem-resistant *E. cloacae* identified in a Chinese gastric cancer patient in 2009 was the first report of an *IMP-1*-producing *Enterobacteriaceae* in China and was found to carry the *blaIMP-1*, *blaCTX-M3*, and *qnrS* genes on three different plasmids [36].

(2) VIM

Verona integron-encoded metallo-β-lactamases (*VIM*) were originally identified in Italy in 1997 [37]. To date, 23 *VIM* variants have been reported, and these enzymes mostly occur in *P. aeruginosa*, though they have also been identified across other bacteria. It was proposed that ceftazidime is a selective pressure that drives the evolution of *VIM*-Type carbapenemases [38]. A recent study that sought to investigate the prevalence of VIM-producing *A. baumannii* from patients with severe UTIs in India found that across 1000 patients, 73 *A. baumannii* isolates were found, of which 34% had detectable *blaVIM* [39]. Another study conducted across hospitals in Egypt found that ~80% of Gram-negative bacilli were found in urine specimens, with *E. coli* being the predominant isolate; 2/3 of the bacterial isolates carried *blaVIM*. Despite their extensive spread across several countries, especially in East India, VIM carbapenemases are relatively rare in the United States. However, a few cases that have emerged across the US have led to a call for national surveillance [40,41].

(3) NDM

New Delhi metallo-β-lactamases (*NDM*) were originally identified in New Delhi, India, in 2009 [42]. Currently, most *NDM* producers are concentrated in Asia, with ~60% of *NDM-1* variants in China and India. In addition, eight variants have been described and identified in this group. NDM genes are dominant in *K. pneumoniae* and *E. coli* isolates. The gene encoding *NDM-1*, a major variant, is often carried by plasmids and, therefore, easily moves to other bacterial species via horizontal gene transfer.

##### Class D Carbapenemases

Class D carbapenemases are serine-β-lactamases that are poorly inhibited by EDTA or clavulanic acid. Most enzymes in this class have been identified in *Acinetobacter* spp., especially in *A. baumannii*.

OXA β-Lactamases

*OXA* β-lactamases are mostly detected in Enterobacteriaceae, *Acinetobacter* spp., and *Pseudomonas* spp. Most OXA-type carbapenemases are encoded by chromosomal genes instead of plasmids and other mobile genetic elements [43]. *OXA-23*, *OXA-24*, *OXA-51* and *OXA-58* are the most common type of *OXA* gene family members which are responsible for carbapenem resistance. Studies have shown that sources of microbes carrying this enzyme can be environmental, such as in municipal wastewater treatment plants, which has implications for the broader dissemination of antibiotic-resistance genes [44]. 

#### 3.1.3. Extended-Spectrum β-Lactamases (ESBLs)

Many drug-resistant urinary pathogens produce extended-spectrum β-lactamases (ESBLs), which break down and destroy commonly used antibiotics, including penicillins and cephalosporins and render them ineffective. ESBLS are a rapidly evolving group of β-lactamases which share the ability to hydrolyze third-generation cephalosporins and aztreonam but are inhibited by clavulanic acid. ESBLs are often encoded by genes located on large plasmids, which also carry genes for resistance to other antimicrobial agents such as aminoglycosides, trimethoprim, sulfonamides, and tetracyclines [45]. In fact, ESBL-producing plasmids have also been shown to carry qnr genes, which mediate fluoroquinolone resistance [46,47]. Thus, very broad antibiotic resistance extending to multiple antibiotic classes is now a frequent characteristic of ESBL-producing enterobacterial isolates.

CTX-M enzymes

The *CTX-M* β-lactamases constitute a rapidly growing family of ESBLs with significant clinical impact and are the most widespread of all ESBLs. In fact, researchers have dubbed this global dissemination the “*CTX-M* pandemic” [48]. *CTX-Ms* are found in at least 26 bacterial species, particularly in *E. coli*, *K. pneumoniae* and *P. mirabilis*. Furthermore, phylogenetic analyses suggest that *CTX-Ms* did not originate from previous plasmid mediated enzymes but through mobilization of chromosomal *bla* genes from *Kluyvera* spp. by transfer through mobile genetic elements [49].

#### 3.1.4. mecA

All methicillin-resistant *S. aureus* (MRSA) contains a copy of a mec gene, most commonly *mecA*. The *mecA* gene is part of a staphylococcal chromosome cassette mec (SCCmec), a mobile genetic element that often contains factors that encode resistance to non-β-lactam antibiotics [50]. *mecA* confers resistance to methicillin and many β-lactams by encoding PBP2A, which has a low binding affinity to most β-lactams [51]. *mecA* does not confer resistance to penicillin G, amoxicillin, ampicillin, ceftobiprole and ceftaroline [52].

### 3.2. Glycopeptide Resistance Genes

Glycopeptides are considered antibiotics of last resort for the treatment of life-threatening infections caused by Gram-positive microbes. This class of drugs targets Gram-positive bacteria by binding to the growing end of peptidoglycan. Glycopeptide-resistant microorganisms use alternative peptidoglycan monomers that result in reduced antibiotic affinity for the cellular target [53].

vanA

The *vanA* gene cluster is the most common mediator of vancomycin resistance in enterococci. Located on Tn1546, *vanA* often resides on a plasmid in vancomycin-resistant enterococci (VRE) [54]. VRE harboring *vanA* can spread rapidly through livestock and remain persistent in the population for decades, even after stopping the use of vancomycin [55,56]. The use of flavophospholipol, an antimicrobial used as a feed additive for livestock, was shown to decrease the horizontal transfer of *vanA* among animals [57].

### 3.3. Macrolides Resistance Genes

ermB

The *ermB* gene confers resistance to macrolides, lincosamides, and streptogramins. *ermB* encodes a methyltransferase that causes ribosomal methylation, resulting in reduced susceptibility to macrolides [58]. In a study that examined the presence of the *ermB* gene in 62 clinical isolates of erythromycin-resistant *S. pneumoniae*, ~60% of which carried *ermB* [59].

### 3.4. Fluoroquinolones Resistance Genes

Fluoroquinolones are broad-spectrum antibiotics that are generally well tolerated due to their high oral bioavailability and large volume of distribution [60]. The most frequently used fluoroquinolones include ciprofloxacin, levofloxacin, norfloxacin, and ofloxacin [61]. Fluoroquinolones target bacterial type II topoisomerases and convert them into cellular toxins [62]. Resistance against fluoroquinolones emerged mainly due to mutations in genes encoding subunits of DNA gyrase and topoisomerase IV and in genes that affect the expression of diffusion channels as well as multidrug-resistance efflux systems [63].

Qnr genes

Plasmid-mediated quinolone resistance is mediated by the qnr genes, composed of the major groups *qnrA*, *qnrB* and *qnrS*. *qnrA* was the first plasmid-mediated quinolone-resistance gene that was identified in a clinical strain of *K. pneumoniae* isolated in 1998 [46]. *qnrB* and *qnrS* have subsequently been observed in other enterobacterial species, including *E. coli*, *Enterobacter* spp., *Salmonella* spp., and *K. pneumonia* [64]. In a study that sought to identify the prevalence of qnr genes among *E. coli* isolated from UTIs of patients in Iran, <90% of isolates tested positive for qnrS by PCR, and ~60% of isolates were resistant to nalidixic acid, a quinolone antibiotic [65].

## 4. Ways to Mitigate Antimicrobial Resistance

Antibiotics have been in use since the early twentieth century and are the most commonly prescribed drugs today. Over the course of the past several decades, however, bacteria have developed AR mechanisms that have led to an ongoing arms race. The rising trend of AR amplifies morbidity, mortality, and economic burden associated with bacterial infections. In addition, the overuse of antibiotics drives the evolution of resistance, as studies have demonstrated a direct relationship between the use of antibiotics and the emergence of resistant bacteria strains [66]. Inappropriate prescribing also significantly contributes to resistant bacteria; incorrect diagnoses or drug regimens were reported in as many as 30% to 50% of cases [67]. In the treatment of uncomplicated UTIs, for example, clinicians often prescribe long-course broad-spectrum antibiotics when only a short course of narrow-spectrum antibiotics is needed [68]. Thus, more conscious prescribing practices are needed among healthcare workers to improve the global burden of AR. Another major contributor to AR is the prophylactic use of antibiotics in animal husbandry. AR genes have been repeatedly shown to jump the species barrier and enter human food sources, which has introduced new resistant bacterial strains [69]. To better regulate this, the World Health Organization (WHO) made the global recommendation to stop the preemptive use of antibiotics in livestock [70]. Collectively, the adoption of these mitigation strategies would significantly improve the incidences of AR bacteria. 

In addition to changes in antibiotic regimens and animal husbandry practices, methods to better identify bacterial genes that confer AR are necessary. The management of bacterial infections in clinical settings, at a minimum, requires accurate detection of antimicrobial resistance in order to guide treatment decisions. Identification of bacterial species alone cannot predict antibiotic susceptibility, which mandates the need for rapid and reliable diagnostic tools to identify AR genes. The preferred method of AR detection by clinical laboratories is culture-based antimicrobial susceptibility testing (AST). However, AST requires a 48–72 h turnaround after specimen collection and is limited to the antimicrobial agents that are included in the panel [71]. These limitations often result in inaccuracies related to antimicrobial susceptibility, which can consequently lead to poor clinical outcomes. One method to counteract this is to apply whole genome sequencing (WGS) for AST (WGS-AST). WGS-AST offers the promise of fast, consistent, and accurate predictions of every known resistance phenotype for a strain [72]. However, while WGS provides significantly more information about bacterial genomes, there is not always phenotypic validation of predictive markers, which leads to poor clinical correlations. Further, costs associated with whole genome sequencing across all patients in a healthcare system will likely be prohibitive. Methods based on polymerase chain reactions (PCR) are effective in identifying known AR genes in a short period of time. PCR is one of the most efficient and rapid molecular tools for the identification and quantification of bacterial AR genes. However, routine surveillance or PCR testing for antibiotic resistance genes among many uropathogens, such as *P. aeruginosa*, is not regularly practiced. Several studies have successfully implemented multiplexed PCRs to simultaneously detect several classes of AR genes from clinical specimens [73,74,75]. Given their cost-effectiveness, ease of use, and rapid turnaround times, multiplexed PCRs provide an excellent diagnostic tool that can assist in determining optimal clinical treatments. Finally, DNA microarrays are an effective method to detect many genes simultaneously in a short time. Numerous microarrays for resistance detection in different species and genera of bacteria have been successfully utilized for clinical specimens [76,77,78]. The limiting factor of diagnostic PCRs and microarrays is only being able to detect known AR genes using designated primers and probes, but these technologies still offer a great deal of valuable information in a short period of time. 

## 5. Conclusions

Antibiotic resistance (ABR) genes exist, thrive, and spread in both nature and humans. Selective pressures imposed by the overuse and misuse of antibiotics aggravate the dissemination of virulent bacterial genes that promote resistance. To alleviate this global burden, clinicians must be more cognizant about prescribing the appropriate treatments at optimal dosages. In the case of UTIs, clinicians should prescribe less broad-spectrum antibiotics when necessary and, in some cases, not prescribe any antibiotics when they are not needed. The use of prophylactic antibiotics in animal livestock must also be decreased to reduce the rapid spread of AR genes. Many AR genes are carried on plasmids, and the ease of conjugative transfer of plasmids allows fast and efficient spread, even across species. In addition, commonly occurring AR genes that encode enzymes such as β-lactamases present many clinical hindrances and force us to constantly develop more antibiotics, each more extreme than the last. As described in Table 1, diagnostic screening of AR genes, such as those that encode ESBLs, *mecA*, and qnr variants via multiplexed PCR or DNA microarrays, is necessary for the determination of the appropriate drug regimens. These methods are also the least financially onerous in hospital settings, do not require extensive training, and provide information that will be used to improve clinical outcomes, which will ultimately better the healthcare landscape and contribute towards antibiotic stewardship.

The incorporation of machine learning technologies for the identification and prediction of antibiotic resistance and susceptibility is on the horizon. Given that plasmid-mediated transfer of ABR genes can transfer between pathogens, machine learning may enable early detection and identification of recurrent UTI infections, novel ABR genes, antibiotic resistance drug profiles, drug avoidance protocols, emerging infectious pathogens, higher risk of disease within certain patient populations, discrete patterns of resistance that may signal global concern, innate metabolic profiles within patients, immunogenicity and drug metabolism profiles. Since antibiotic resistance and stewardship are predicated on the detection of ABR genes, understanding the incidence, frequency and distribution for global public health is important to combat the evolution of ABR genes. In addition, the development of novel antibiotics to prevent the accumulation of ABR mechanisms will be important. Future work may include exploratory analysis for predictions of infectious states in UTIs based on ABR gene detection in addition to novel drug discovery for antibiotic targets. 

Considerations of the polymicrobial nature of UTIs further enhance the gene transfer mechanisms within genera and species of pathogens. Traditionally, UTI diagnostics involve urine culture followed by AST and minimum inhibitory concentration (MIC) testing. However, we know now that the polymicrobial nature of UTIs can potentially explain the recurrent nature of antibiotic-resistant pathogens, given that pathogens are able to share metabolites. Therefore, products of antibiotic resistance genes (inclusive of the genes via plasmids and mobile genetics elements) can create a complex architecture of resistance mechanisms that may confound clinician-guided treatment decisions. A thorough understanding of the ABR genes that are involved can help in treatment decisions of current infectious states while enabling predictive and preventative insights for future infections. Rapid and early detection of ABR genes is important, especially to guide antibiotic drug avoidance protocols whereby the clinician can avoid overprescription practices of antibiotics that will have no meaningful effect due to resistance. Enabling the stratification of ABR genes can further allow clinicians to take targeted therapeutic antibiotic approaches as opposed to broad-spectrum treatments. Overall, this increases the precision medicine aspects of antibiotic administration while incorporating judicious antibiotic stewardship practices. In addition to diagnostic models, it is equally important to implement routine monitoring paradigms to track the evolution of antibiotic resistance over time. Future work will explore targeted and massively multiplexed detection of antibiotic resistance genes that cover a wide variety of antibiotic classes via a rapid molecular diagnostic assay for UTI patient stratification. Such work will benefit clinician decision-making to implement targeted antibiotic therapy practices and clinical stratification of the various segments within complicated vs. uncomplicated UTIs and symptomatic vs. asymptomatic UTIs. Collectively, ABR gene detection is important for both diagnostics and monitoring in multiple infectious disease models.

## Figures and Tables

**Table 1:** Overview of bacterial antibiotic resistance mechanisms for uropathogens.

Resistance Mechanism/Enzyme	Examples Genes	Prominent Example Uropathogenic Organism(s)	Example Resistance Profile
β-lactamases	*CMY-2*, *FOX*	*Enterobacteriaceae*	penicillins, second and third-generation cephalosporins and cephamycins
Carbapenemases	*blaKPC*	*Klebsiella pneumoniae*	penicillins, cephalosporins, monobactams, and carbapenems
	*IMP*, *VIM*, *NDM*	*Enterobacteriaceae*, *P. aeruginosa*	carbapenems, penicillins, carbapenems (varies)
	*OXA*	*Acinetobacter* spp. (especially *A. baumannii*)	penicillins, cephalosporins (varies), carbapenems,
ESBLs	*CTX-M*	*Enterobacteriaceae*	penicillins, cephalosporins, monobactams
mecA	*mecA*	Methicillin-resistant *S. aureus* (MRSA)	methicillins and several other β-lactams
Glycopeptide resistance genes	*vanA*	*Enterococci*	vancomycin
Macrolide resistance genes	*ermB*	*Enterobacteriaceae*	macrolides, lincosamides, and streptogramins
Fluoroquinolone resistance genes	*qnr*	*Enterobacteriaceae*	fluoroquinolones
